# Transosseous versus transmuscular repair of the posterior soft tissue in primary hip arthroplasty: a meta-analysis

**DOI:** 10.1186/s13018-020-02084-9

**Published:** 2020-11-19

**Authors:** Changjiao Sun, Xiaofei Zhang, Qi Ma, Ruiyong Du, Xu Cai, Huadong Yang

**Affiliations:** 1 Department of Orthopedic, Beijing Tsinghua Changgung Hospital, School of Clinical Medicine, Tsinghua University, No.168 Litang Road, Dongxiaokou Town, Changping District, Beijing, 102218 China; 2 Department of Clinical Epidemiology and Biostatistics, Beijing Tsinghua Changgung Hospital, School of Clinical Medicine, Tsinghua University, No.168 Litang Road, Dongxiaokou Town, Changping District 102218, Beijing, China

**Keywords:** Transosseous, Transmuscular, Dislocation, Bone to tendon, Tendon to tendon, Total hip arthroplasty, Posterolateral approach

## Abstract

**Background:**

During the posterior approach, it has been shown that a significant reduction in dislocation rate can be achieved with the repair of the posterior soft tissue. However, no consensus exists about the best way to perform this repair. This review aimed to compare the transosseous with transmuscular repair of the posterior soft tissue in total hip arthroplasty (THA).

**Methods:**

We conducted a meta-analysis to identify studies involving transosseous versus transmuscular repair of the posterior soft tissue in THA in electronic databases, including Web of Science, Embase, PubMed, Cochrane Controlled Trials Register, Cochrane Library, Highwire, CBM, CNKI, VIP, Wanfang database, up to July 2020. Finally, we identified 1417 patients (1481 hips) assessed in seven studies.

**Results:**

Compared with transmuscular repair, transosseous repair resulted in less incidence of dislocation (*P* = 0.003), less blood loss during operation (*P* < 0.00001) and lower VAS score within 3 months (*P* = 0.02). There were no significant differences in terms of trochanteric fracture rate (*P* = 0.56), Harris hip score at 3 months (*P* = 0.35) and 6 months (*P* = 0.89), VAS score within 6 months (*P* = 0.53), and operation time (*P* = 0.70) between two groups.

**Conclusion:**

The lower dislocation rate, less blood loss, and lower VAS scores after operation supported transosseous repair's superiority to transmuscular repair. Besides, no additional medical cost and operating time were associated with transosseous repair compared with transmuscular repair. Hence, we recommend that transosseous repair be chosen first by orthopedists when performing reconstruction of the posterior soft tissue in THA via a posterolateral approach. Given the relevant possible biases in our meta-analysis, we required more adequately powered and better-designed RCT studies with long-term follow-up to reach a firmer conclusion.

## Introduction

Several surgical approaches have been implemented in total hip arthroplasty (THA). Among them, the posterolateral approach serves as the most common approach mainly for its minimal trauma to the hip abductors, diminished blood loss, less operative time, excellent visualization of both the acetabulum and proximal femur, and small learning curve [1–6]. However, the hip dislocation rate associated with the posterolateral approach is approximately 2.1–7.5%, higher than the 0–2.3% rate for the trans-trochanteric or anterolateral approach [7, 8], which is attributed to posterior capsule and short-external rotator muscle damages [9].

The posterior approach showed that the postoperative dislocation rate was reduced when the posterior capsule and external rotators were carefully repaired in THA via a posterolateral approach [10–12]. In their meta-analysis, Kwon et al. found a relative risk of dislocation eight times greater if no soft tissue repair was performed [13]. However, no consensus exists about the best way to perform this repair. The repair techniques are mainly divided into two categories: (1) transosseous(tendon-to-bone)repair, in which the short external rotator tendon with the posterior capsule is reattached to the greater trochanter transosseous [5, 11, 14–16] and (2) transmuscular (tendon-to-tendon) repair, in which the posterior soft tissue flap is reattached directly to the gluteus medius tendon [4–6, 11, 16–19].

Although previous studies provided adequate reported outcomes of transosseous and transmuscular repair, there is no consensus and evidence-based medicine on the best repair technique of posterior soft tissue (PST). Therefore, in this meta-analysis, our specific purpose is to comprehensively compare transosseous and transmuscular posterior soft tissue repair and give more evidence-based data for orthopedists.

## Methods

The current meta-analysis was registered on PROSPERO (International prospective register of systematic reviews) and the registration number was CRD42020199698.We strictly followed the PRISMA (preferred reporting items for systematic review and meta-analysis) guidelines to conduct this analysis [20] according to the preferred reporting items for systematic reviews and meta-analyses statement.

### Search strategy

We conducted a meta-analysis to identify studies involving transosseous and transmuscular repair of the posterior soft tissue in total hip arthroplasty in electronic databases, including Web of Science, Embase, PubMed, Cochrane, Controlled Trials Register, Cochrane Library, Highwire, CBM, CNKI, VIP, Wanfang database, up to July 2020. The keywords used were “total hip arthroplasty,” “total hip replacement,” “posterior soft tissue repair,” “posterior capsule repair,” “Transosseous,” “transmuscular,” “tendon-to-bone” “tendon-to-tendon,” “dislocation” in conjunction with Boolean operators “AND” or “OR.” Review Manager Software was used to perform the meta-analysis.

### Inclusion criteria

All randomized controlled trials (RCTs) and non-randomized controlled trials (nRCTs) comparing transosseous and transmuscular repair of the posterior soft tissue in primary THA were identified and included from the search strategy. These studies should meet the following inclusion criteria: (1) The THA procedure was performed for the first time. (2) Transosseous repair of the posterior soft tissue was involved. (3) The comparator was transmuscular repair of the posterior soft tissue in the original comparative study. (4) A posterolateral or posterior approach was used. (5) At least one of the following indexes was reported: dislocation rate, trochanteric fracture, Harris hip score, VAS score, blood loss during operation, and operation time. We also excluded: (1) studies that revision of THA was performed, (2) unclear or incomplete sample data were available, (3) posterior capsule was not mentioned during the procedure, (4) the anterior approach was used, and (5) the postoperative effect was inaccurate.

### Data extraction process

All randomized controlled trials (RCTs) and non- randomized controlled trials (n RCTs) comparing transosseous repair of the posterior soft tissue and transmuscular repair of the posterior soft tissue with primary THA were identified and included from the search strategy. Two researchers independently scanned the titles and abstracts of all literature searched, and they independently extracted the available data from each study. After excluding the trials which did not meet the inclusion criteria, we read the full text of the literature that might meet the inclusion criteria to determine whether this literature ultimately met the inclusion criteria. In the process of cross-checking, disagreements were resolved by discussion to reach consensus. Data were extracted based on the following: (1) research features (i.e., authors, type of study, year of publication, and country), (2) population information (i.e., gender, body mass index [BMI], age follow-up time, and (3) intervention (i.e., diagnosis, the diameter of the femoral head, cup position, diameter of drilled holes). If the necessary results are omitted, we will contact the authors by email or other means to obtain more data if necessary

### Assessment of studies

To assess the methodological quality, we evaluated the nonrandomized studies using the nine-star Newcastle-Ottawa scale (NOS), a validated tool suitable for assessing the quality of nonrandomized studies [21]. According to the Cochrane Handbook for Systematic Reviews of Interventions, the methodological quality and basis of the RCTs were assessed as follows: randomization, allocation concealment, blind method, selective reporting, group similarity at baseline, incomplete outcome data, compliance, timing of outcome assessments, and intention-to-treat analysis [22]. Two researchers independently assessed the studies, and disagreements between them were resolved through discussions with a third author or consensus.

### Statistical analysis

The *I*^2^ and *Q* test was used to evaluate the heterogeneity between studies. *P* values ≤ 0.1 or *I*^2^ value> 50% suggested a high degree of heterogeneity; thus, we used the randomized-effects model. Otherwise, we used the fixed-effects model [20]. In each study, the odds ratio (OR) and relevant 95% confidence interval (CI) were commonly used to measure dichotomous variables such as rates of postoperative dislocation and trochanteric fracture. Given that the outcome is rare, reported OR was supposed to approximate RR (relative risk) based on Cornfield’s rare disease outcome assumption [23]. The mean difference (MD) or standard MD was used to assess continuous outcomes such as blood loss during operation, Harris hip score, VAS score, and operation time with a 95% confidence interval (CI). We used statistical algorithms to estimate the standard deviation for those studies that provided only continuous variables for means and range [24]. If *P* values were less than 0.05, we considered the results as a statistically significant difference. Sensitivity analysis was used to assess the stability of the results (if necessary), and subgroup analysis was conducted to get more specific and detailed results if the data were available. All statistical analyses were performed using Review Manager (version 5.3 for MAC, the Cochrane Collaboration, the Nordic Cochrane Centre, Copenhagen, 2014).

## Results

### Search results

**Fig. 1:**
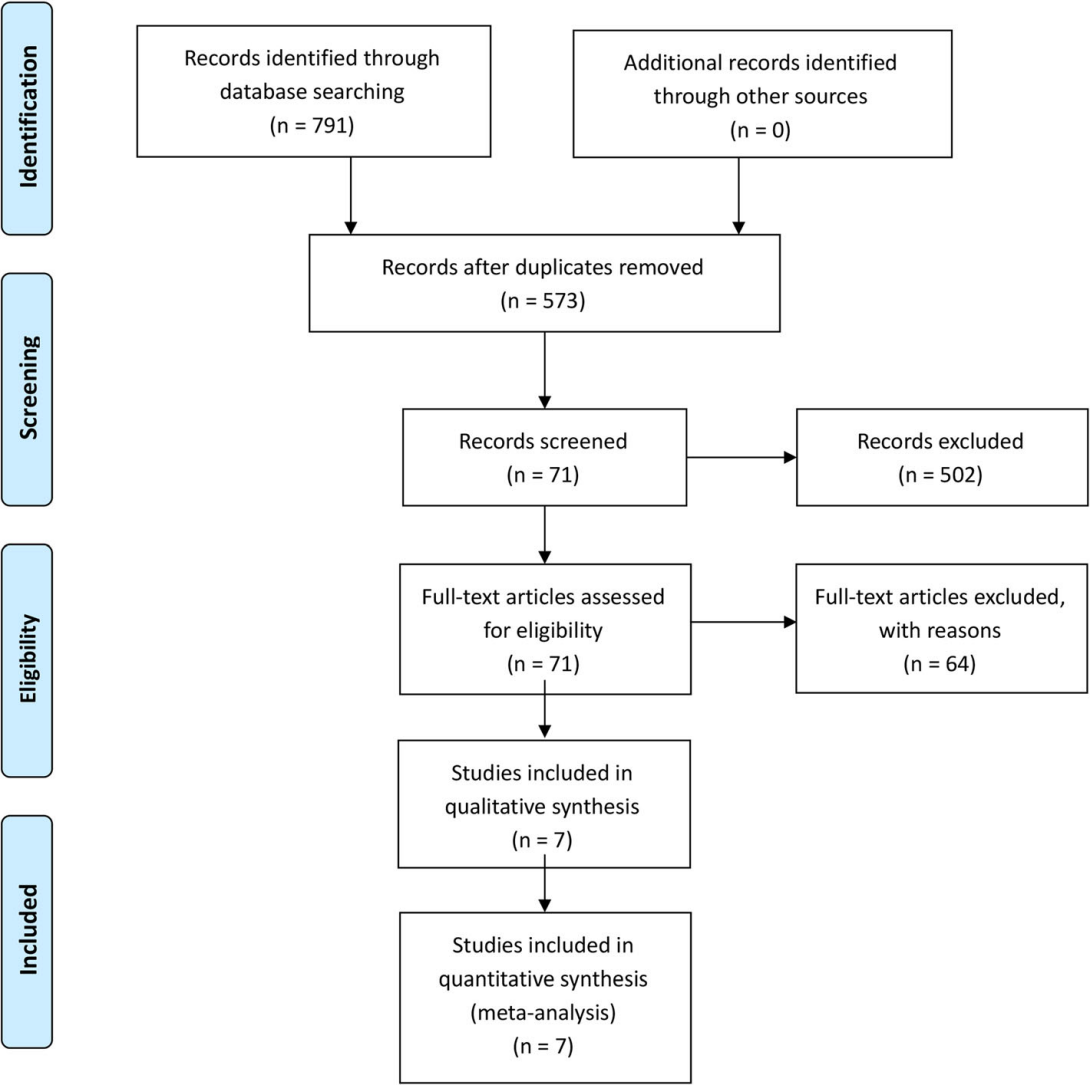
The search results and selection procedure. Seven hundred ninety-one relevant citations were identified from the databases according to the literature search strategy described earlier. After deleting 218 duplicates, we obtained 573 articles. Upon review of titles and abstracts of the 573 remaining articles, 502 irrelevant clinical studies were excluded. By reading the 71 full-text articles, we excluded another 64 articles for the following reasons such as (systematic) reviews, surgery techniques, none-compare groups, cadaver researches, animal researches, and no useful outcome data. The remaining seven articles were deemed appropriate. Finally, we identified 1417 patients (1481 hips) assessed in (2 RCTs [30, 31] and 5 nRCTs

The literature search and selection process are shown in Fig. 1. Finally, seven publications [25–31] from 2012 to 2019 were included in our meta-analysis. The detailed literature screening process is shown as the PRISMA flow diagram in Fig. 1. Seven hundred ninety-one relevant citations were identified from the databases according to the literature search strategy described earlier. After deleting 218 duplicates, we obtained 573 articles. Upon review of titles and abstracts of the 573 remaining articles, 502 irrelevant clinical studies were excluded. By reading the 71 full-text articles, we excluded another 64 articles for the following reasons such as (systematic) reviews, surgery techniques, none-compare groups, cadaver researches, animal researches, and no useful outcome data. The remaining seven articles were deemed appropriate. Finally, we identified 1417 patients (1481 hips) assessed in (2 RCTs [30, 31] and 5 nRCTs [25–29])

### Study characteristics and quality

The detailed baseline characteristics and general intervention information are presented in Tables 1, 2, and 3. All the articles were published in English and Chinese between the years 2012 and July 2020.

**Table 1:** Summary of studies characteristics

Author	Year	Country	Design	Follow-up time (transosseous /transmuscular) (months)
**Cai et al.**	2013	China	Retrospective study	6/6
**Cao et al.**	2012	China	Retrospective study	6/6
**Liu et al.**	2018	China	Retrospective study	6/6
**Moon et al.**	2018	South Korea	RCT	28/29
**Wu et al.**	2019	China	Retrospective study	6/6
**Shen et al.**	2015	China	Retrospective study	19.74/22.37
**Spaas et al.**	2015	Netherlands	RCT	12/12

*Abbreviations*: *RCT* randomized controlled trials. Summary of studies characteristics including a year of publication, country, design, and follow-up time

**Table 2:** Summary of patient demographic details for each study

Author	Patients	THAs	Age (years)	Female (%)	BMI (kg/m^**2**^)
**Cai et al.**	164/98	213/104	71.8/66.3	56.1/57.1	NA
**Cao et al.**	30/30	30/30	NA	NA	NA
**Liu et al.**	40/40	6/6	74.5/74.7	47/50	NA
**Moon et al.**	85/73	87/80	69/72	72.4/63.8	24.7/24.1
**Wu et al.**	46/41	46/41	57.7/60.9	56.5/53.7	26 /25
**Shen et al.**	126/179	126/179	51.55/53.32	40.5/40.2	NA
**Spaas et al.**	219/246	219/246	71/69	64/66	NA

*Abbreviations*: *BMI* body mass index. Summary of patient demographic information for each study including number of THAs/patients, gender, age, and BMI

**Table 3:** The detailed characteristics of general intervention information

Author	Cai et al.	Cao et al.	Liu et al.	Moon et al.	Wu et al.	Shen et al.	Spaas et al.
**Transosseous /transmuscular**							
**Diagnosis**							
**Osteonecrosis of femoral head**	0/0	NA	NA	51/46	27/23	20/34	0/0
**Hip osteoarthritis**	213/104	NA	NA	35/32	7/2	89/124	219/246
**Femoral neck fracture**	0/0	NA	NA	0/0	7/13	0/0	0/0
**Developmental dysplasia of hip**	0/0	NA	NA	0/0	5/3	0/0	0/0
**Traumatic arthritis**	0/0	NA	NA	0/0	0/0	6/13	0/0
**Rheumatoid arthritis**	0/0	NA	NA	1/2	0/0	11/8	0/0
**Femoral head (mm)**				32 or 36			
**24**	NA	NA	NA		0/1	0/0	0/0
**28**	NA	NA	NA		2/6	126/179	0/0
**32**	NA	NA	NA		16/18	0/0	0/0
**36**	NA	NA	NA		25/15	0/0	219/246
**40**	NA	NA	NA		3/1	0/0	0/0
**Cup position**							
**Anteversion (°)**	NA	NA	NA	20.2/19.5	14/14.3	14.51/14.56	NA
**Abduction (°)**	NA	NA	NA	42.5/41.7	36.5/37	44.97/45.21	NA
**Diameter of drilled holes (mm)**	NA	2.3	2	NA	2	2	2.3
**Tranexamic acid use**	NA	NA	NA	NA	Yes	NA	NA

The detailed characteristics of general intervention information, including diagnosis, the diameter of the femoral head, cup position, diameter of drilled holes, tranexamic acid use

### Risk of bias assessment

The methodological quality of the involved studies ranged from 6 to 8 (Table 4). The risk of bias summary and risk of bias graph for RCTs are shown in Table 5. As a result, the overall quality of the included studies was considered adequate.

**Table 4:** Risk-of-bias assessment for the studies included in the meta-analysis (NOS)

(nRCT) Study = 6	Selection				Comparability	Outcome/exposure			Score
	Item 1	Item 2	Item 3	Item 4	Item 5	Item 6	Item 7	Item 8	
**Cai 2012**	*		*	*	*	*		*	**6**
**Liu 2018**	*		*	*	*	*		*	**6**
**Nmed 2019**	*	*	*	*	*	*		*	**7**
**Shen 2015**	*	*	*	*	*	*		*	**7**
**Wu 2019**	*	*	*	*	*	*		*	**7**
**Cao 2012**	*	*	*	*	*	*	*	*	**8**

The methodological quality of the involved studies ranged from 6 to 8

**Table 5:** Methodological assessment according to six domains of potential biases (Cochrane Risk of Bias Tool)

RCT Study = 2	Random sequence generation	Allocation concealment	Blinding of participants and personnel	Blinding of outcome assessment	Incomplete outcome data	Selective reporting	Other bias
**Moon 2018**	Low	Low	Low	Low	Low	Low	Unclear
**Spaans 2015**	High	High	Unclear	Low	Low	Low	Unclear

*Abbreviations*: *RCT* randomized controlled trials

The RCTs' methodological quality and basis were assessed as follows: randomization, allocation concealment, blind method, selective reporting, group similarity at baseline, incomplete outcome data, compliance, the timing of outcome assessments, and intention-to-treat analysis

### Postoperative dislocation rate

**Fig. 2:**
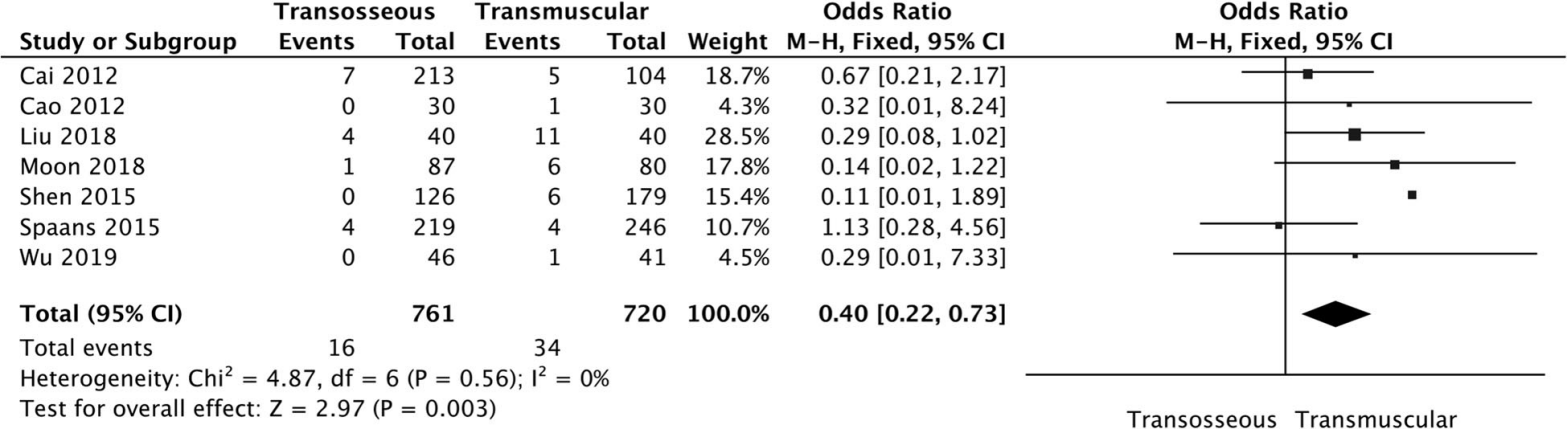
A forest plot diagram showing the incidence of dislocation. Seven studies assessed the incidence of dislocation. Heterogeneity test (*I*^2^ =0%) indicated that there is statistical homogeneity among the studies, so the fixed-effect model was used to analyze. The results of the meta-analysis showed that there was a significant difference in the incidence of postoperative dislocation between transosseous repair group and transmuscular repair group (OR 0.4, 95% CI 0.22–0.73, *P* = 0.003, *I*^2^ = 0%)

Seven studies assessed the incidence of dislocation. The heterogeneity test (*I*^2^ = 0%) indicated statistical homogeneity among the studies, so the fixed-effect model was used to analyze. The meta-analysis results showed that there was a significant difference in the incidence of postoperative dislocation between the transosseous repair group and the transmuscular repair group (OR 0.4, 95% CI 0.22–0.73, *P* = 0.003, *I*^2^ = 0%; Fig. 2). To ensure the accuracy and stability of the study, we continued to carry out sensitivity analysis. The sensitivity analysis of the seven works of the literature showed that none of them interfered with the meta-analysis results, which meant this study had excellent stability. The results indicated that compared with transmuscular repair of posterior soft tissue, the transosseous repair is more helpful to reduce the incidence of hip dislocation after primary THA.

### Trochanteric fracture

**Fig. 3:**
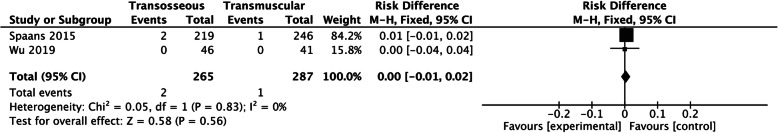
A forest plot diagram showing Trochanteric fracture rate. Two studies reported the Trochanteric fracture rate. The results showed no significant statistical difference between the 2 groups (RD 0.00, 95% CI − 0.01 to 0.02, *P* = 0.56, *I*^2^ = 0%)

Two studies reported the Trochanteric fracture rate. The results showed no significant statistical difference between the two groups (RD 0.00, 95% CI − 0.01 to 0.02, *P* = 0.56, *I*^2^ = 0%; Fig. 3). According to the heterogeneity test in this study (*P* = 0.83, *I*^2^ = 0%), it was suggested that there was statistical homogeneity among the selected literature in this study, so we chose the fixed-effect model for meta-analysis.

### Harris hip score (HHS)

**Fig. 4:**
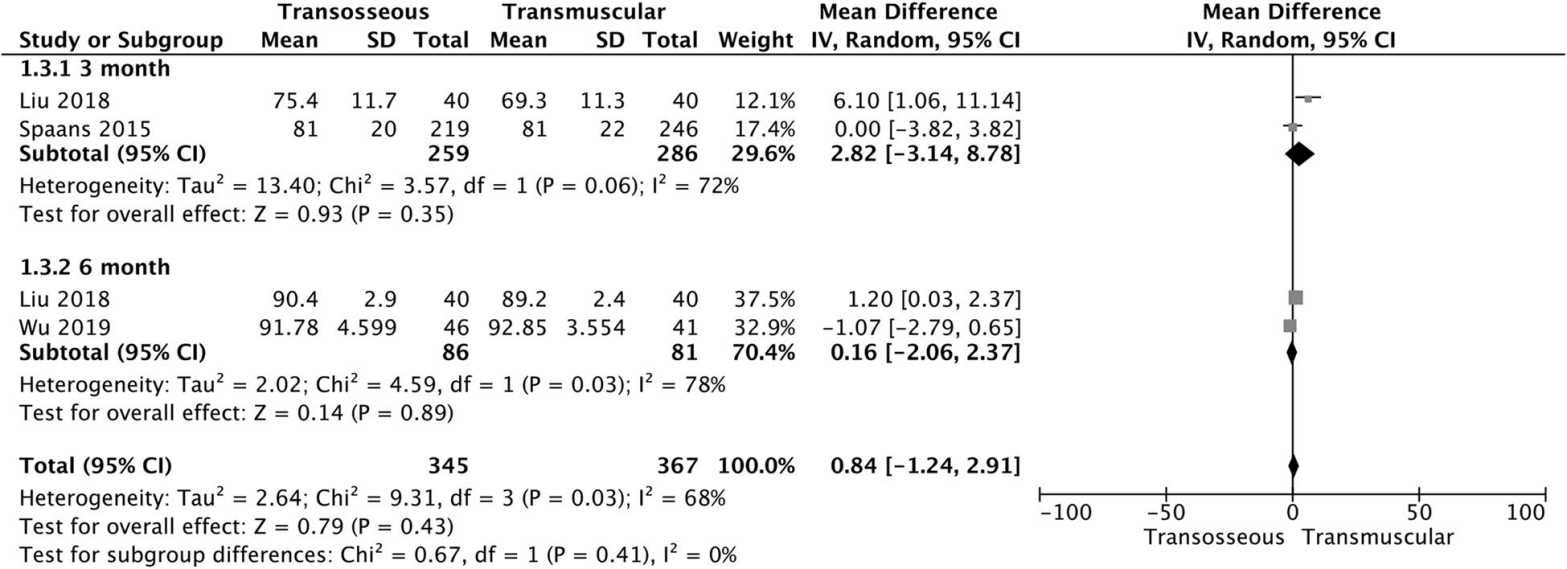
A forest plot diagram showing Harris hip score (HHS). Two studies reported the Harris hip score (HHS) at 3 months and two studies reported the HSS at 6 months, and the results showed no significant statistical difference between the 2 groups at 3 months (MD 2.82, 95% CI − 3.14 to 8.78, *P* = 0.35, *I*^2^ = 72%) and at 6 months (MD 0.16, 95% CI − 2.06 to 2.37, *P* = 0.89, *I*^2^ = 78%)

**Fig. 5:**
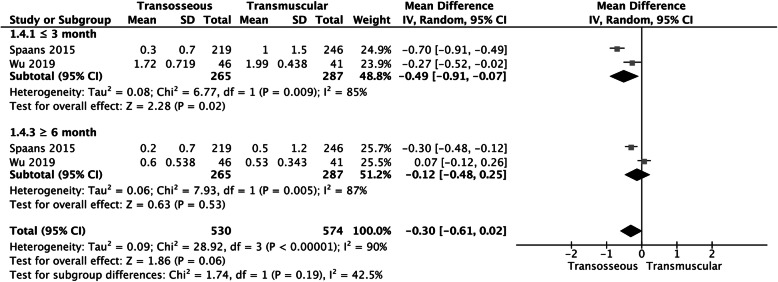
A forest plot diagram showing VAS score. Two studies reported the VAS score within 3 months and 6 months. The results showed significant statistical difference between the 2 groups within 3 months (MD − 0.49, 95% CI − 0.91 to − 0.07, *P* = 0.02, *I*^2^ = 85%; Fig. 6) and no significant statistical difference between the 2 groups within 6 months (MD − 0.12, 95% CI − 0.48 to 0.25, *P* = 0.53, *I*^2^ = 87%)

Two studies reported the Harris hip score (HHS) at 3 months, and two studies reported the HSS at 6 months, and the results showed no significant statistical difference between the two groups at 3 months (MD 2.82, 95% CI − 3.14 to 8.78, *P* = 0.35, *I*^2^ = 72%; Fig. 4), and at 6 months (MD 0.16, 95% CI − 2.06 to 2.37, *P* = 0.89, *I*^2^ = 78%; Fig. 5) According to the heterogeneity test in this study (*P* = 0.06, *I*^2^ = 72%), (*P* = 0.03, *I*^2^ = 78%), it was suggested that there was heterogeneity among the selected literature in this study, so we chose the random effect model for meta-analysis.

### VAS score

Two studies reported the VAS score within 3 months and 6 months. The results showed significant statistical difference between the 2 groups within 3 months (MD − 0.49, 95% CI − 0.91 to − 0.07, *P* = 0.02, *I*^2^ = 85%; Fig. 5) and no significant statistical difference between the 2 groups within 6 months (MD − 0.12, 95% CI − 0.48 to 0.25, *P* = 0.53, *I*^2^ = 87%; Fig. 5) According to the heterogeneity test in this study (*P* = 0.009, *I*^2^ = 85%), (*P* = 0.005, *I*^2^ = 87%), it was suggested that there was heterogeneity among the selected literatures in this study, so we chose the random effect model for meta-analysis.

### Operation time

**Fig. 6:**
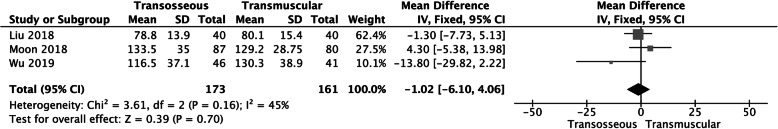
A forest plot diagram showing operation time. Three studies reported the operation time. The results showed no significant statistical difference between the 2 groups (MD − 1.02, 95% CI − 6.1 to 4.06, *P* = 0.70, *I*^2^ = 45%)

Three studies reported the operation time. The results showed no significant statistical difference between the 2 groups (MD − 1.02, 95% CI − 6.1 to 4.06, *P* = 0.70, *I*^2^ = 45%; Fig. 6). According to the heterogeneity test in this study (*P* = 0.16, *I*^2^ = 45%), it was suggested that there was statistical homogeneity among the selected literature in this study, so we chose the fixed-effect model for meta-analysis.

### Blood loss during operation

**Fig. 7:**
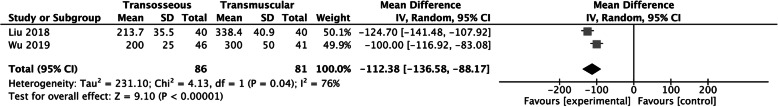
A forest plot diagram showing blood loss during operation. Two studies reported the blood loss during operation. The results showed a significant statistical difference between the two groups (MD − 112.38 95% CI − 136.58 to − 88.17, *P* < 0.00001, *I*^2^ = 76%)

Two studies reported the Blood loss during operation. The results showed a significant statistical difference between the two groups (MD − 112.38 95% CI − 136.58 to − 88.17, *P* < 0.00001, *I*^2^ = 76%, Fig. 7). According to the heterogeneity test in this study (*P* < 0.00001, *I*^2^ = 76%), it was suggested that there was heterogeneity among the selected literature in this study, so we chose the random effect model for meta-analysis. The results indicated that compared with transmuscular repair of posterior soft tissue, transosseous repair has less blood loss during operation.

## Discussion

Although uncommon, postoperative hip dislocation after arthroplasty is a complication with serious consequences. The chance of dislocation is influenced by multiple factors [1, 32–37], which could be mainly divided into three aspects : (1) patient-related factors, (2) surgery related factors, and (3) postoperative management factors. Patient-related factors include age(> 80 years) [38], gender(female), original diseases such as mental illness, neuromuscular disease, Parkinson’s disease, and surgery history of the hip [39]. Surgery-related factors included surgery approach [40], surgeon experience [17], prosthesis position [40], offset [41], femoral head diameter [42], prosthesis design [43], and soft tissue repair [12, 44]. Postoperative management was also important for patients after THA. Patients should be informed to avoid doing high-risk postures [45].

Of these factors, surgeons can control surgical-related factors. In the posterolateral or posterior approach, which may induce posterior capsule and short external rotator muscle damage increasing the patient’s vulnerability to postoperative dislocation [46]. Researchers have attempted to identify methods for reducing the postoperative dislocation rate by repairing the posterior structures, including the posterior capsule and short external rotator tendon.

Three meta-analyses [12, 44, 47] found a relatively higher risk of dislocation more significant if no soft tissue repair was performed. There are two ways of posterior repair of soft tissue. Some surgeons performed a transosseous repair of soft tissue by drilling holes [14–16, 48]. Several authors performed soft tissue repair without drilling holes [17–19]. Both ways of repair showed decreasing dislocation rates [12, 44].

Some authors [25, 30] compared the dislocation rate between transosseous and transmuscular repair and reported that transosseous and transmuscular repair were equally effective techniques without significant differences in dislocation rates. They believed that transmuscular repair was easy to perform, and the elastic suture site had the potential to avoid tearing of the reconstructed posterior soft tissue during healing. Drilling holes could be 1 of the factors causing a longer operation time in the transosseous group. Passing sutures through the trochanter by drilled holes can locally weaken the bone, which may cause the failure of the repair and trochanteric avulsion fractures [6]. However, some authors believe transosseous reapproximation of the capsule and/or short external rotators to the posterior aspect of the greater trochanter through drilled holes in the bone is thought to give better strength and probably a more anatomical repair [11, 14–16]. Moon et al. [31] reported that suture failure was observed less frequently in the transosseous group (18.4%) than in the transmuscular repair group (65%). The dislocation rate was significantly higher in the transmuscular repair group compared with the transosseous group.

It considered the uncertainty and controversy about the influence of transosseous and transmuscular posterior soft tissue repair on the incidence of hip dislocation following primary THA. We sought to evaluate the body of evidence linking transosseous and transmuscular repair with the risk of hip dislocation following primary THA carrying out a comprehensive systematic review of RCTs and observational studies. To our knowledge, this is the first meta-analysis comparing the transosseous repair and transmuscular repair of posterior soft tissue in primary THA. This meta-analysis included seven studies (2 RCTs and five nRCTs) that analyzed 1417 patients (1481 hips) and compared the transosseous repair group’s clinical effectiveness and the transmuscular repair group. Lower dislocation rate, less blood loss during operation, and lower pain scores within 3 months supported the superiority of transosseous repair to transmuscular repair based on the existing evidence base. There was no significant difference in trochanteric fracture rate, operation time, HHS score, and VAS score within 6 months between two groups.

In a biomechanical study, Sioen et al. [49] revealed that the mechanical strength of transosseous repair was superior to that of transmuscular repair. They measured the torsion strength and rotation angle of the hip under three repair conditions, including no repair, transosseous repair, and transmuscular repair. They found that the transosseous repair group’s torsion strength was four times as much as that when the repair was not done and twice that of the transmuscular repair group. The transosseous repair group's hip rotation angle was increased by 83% compared to that in the no repair group and by 46% in the transmuscular group. Moon et al. [31] provided reliable clinical data for transosseous and transmuscular repair. They compared the rates of repair failure after the two repair techniques. Of the 167 hips included, 87 hips underwent transosseous repair, and 80 hips underwent transmuscular repair. Suture failure was observed less frequently in the transosseous repair group (18.4%) than in the transmuscular repair group (65%). These biomechanical features and the low-frequency suture failure of the transosseous repair group, seem to contribute to the decreased dislocation rate in transosseous repair group more than the transmuscular repair group in our meta-analysis

Zhou et al. [47] reported that drilling holes in the greater trochanter would reduce bone strength and increase fracture risk. Intraoperative periprosthetic fractures are also mentioned as a rare complication of uncemented total hip replacement. However, our data showed there were no significant differences in terms of the trochanter fracture rate. White et al. [6] revealed that when 2.7-mm bone holes reconstructed the posterior, the greater trochanteric fracture was 0.9%. They believed that greater trochanteric fractures could be avoided by reducing the diameter of bone holes. Osmani et al. [50] reduced the holes to 2.3 mm, and no greater trochanteric fracture was observed in 150 patients during follow-up. The diameter of the bone holes in our data is no more than 2.3. We believed that reasonable holes had no significant effect on bone strength, and the bone holes were strong enough to resist the pull of the sutures.

Some surgeons believe transosseous repair with drilling holes may cause a longer operation time. However, no significant difference was observed in terms of operation time between the two groups in our meta-analysis. Multiple factors influence the operation time. More prospective randomized trials investigating operation is needed to confirm the results.

As for blood loss, our data showed less blood loss during operation in the transosseous repair group. The posterior soft tissue flap is reattached directly to the gluteus medius tendon with the transmuscular repair method. This tendon to tendon or muscle repair may cause more blood loss during the operation.

Harris hip score is frequently used to assess the outcome and effectiveness of total hip arthroplasty. In our meta-analysis, no significant difference was observed in terms of HSS at 3 months and 6 months between two groups. We believe that the main advantage of posterior reconstruction, either with the transosseous repair or transosseous is the reconstruction of biofeedback. The patient will notice that too much internal rotation of the hip could lead to dislocation. In that objective, it would seem logical that the HSS of transmuscular and transosseous repair is comparable.

Our data also showed that the VAS scores within 3 months after surgery were significantly lower in the transosseous group than those in the transmuscular group. The difference disappeared within 6 months after surgery. According to these results, transosseous repair appears to present superiority in early pain. We think the reason may be that the posterior soft tissue was displaced from their normal anatomical position with transmuscular repair. Additional strain in the reconstructed soft tissue would occur when the affected hip rotated internally, which might generate the difference in early pain between the two repair techniques.

The limitations of this study are as follows. Firstly, we only included two randomized controlled trials; the other five studies were observational studies, which may have reduced the quality of the evidence for this meta-analysis. Although we have included all related studies thus far and tried to collect more data to make this meta-analysis and assess its effect, more prospective randomized trials investigating dislocation incidence and other clinical parameters are needed to confirm the results and conclusions. Secondly, the follow-up time was short that long-term consequences concerning transosseous repair and transmuscular repair were unknown. Thirdly, there is diversity in the size of the femoral head, and the detailed data is not clear in most included articles. We cannot perform a subgroup analysis to see if the dislocations rates differed with studies reporting larger head sizes versus smaller head sizes. Fourth, many other factors affect the rate of dislocation of a THA. There is diversity in the follow-up time, patient factors, surgeon factors, and so on, so the heterogeneity is increasing among included studies.

## Conclusion

In conclusion, the lower dislocation rate, less blood loss, and lower VAS score early after operation supported the superiority of transosseous repair to transmuscular repair. Besides, no additional medical cost and operating time were associated with transosseous repair compared with transmuscular repair. Hence, we recommend that transosseous repair could be chosen first by orthopedists when performing reconstruction of the posterior soft tissue in THA via a posterior or posterolateral approach. Given the relevant possible biases in our meta-analysis, more adequately powered and better-designed RCT studies with long-term follow-up were required to reach a firmer conclusion.

## Data Availability

The datasets generated during and/or analyzed during the current study are available from the corresponding author on reasonable request.

## Abbreviations

CIs: Confidence intervals
RCTs: Randomized controlled trials
RR: Risk ratio
OR: Odds ratio
VMD: Weighted mean difference
THA: Total hip arthroplasty
BMI: Body mass index
